# Supplementary material for: Evaluation of dynamic cerebrovascular autoregulation during liver transplantation
Source: PLoS One. 2024 Jul 26;19(7):e0305658. doi: 10.1371/journal.pone.0305658 (PMC11280153; doi:10.1371/journal.pone.0305658)
Supplement: S1 Table — (DOCX) [file pone.0305658.s001.docx]

**Table S1. Clinical data of all 20 Patients undergoing liver transplantation.**

| Patient | Age (yrs.) | Sex | ASA | MELD  Score | Dialysis | HE | Aetiology | Onset | Delirium  (within 4 days at ICU) | One-year mortality |
| --- | --- | --- | --- | --- | --- | --- | --- | --- | --- | --- |
| 1 | 31 | M | 2 | 6 | no | no | HCC | CLF | no | no |
| 2 | 57 | F | 2 | 30 | no | no | Cryptogenic cirrhosis | ACLF | no | no |
| 3 | 56 | F | 3 | 19 | no | no | PBC, autoimmune hepatitis | CLF | no | no |
| 4 | 59 | F | 4 | 14 | no | yes | NASH | CLF | no | yes |
| 5 | 64 | M | 3 | 10 | no | yes | NASH | CLF | no | no |
| 6 | 66 | M | 4 | 11 | no | no | IgG4-related SC | CLF | no | yes |
| 7 | 38 | M | 3 | 34 | no | no | Hepatitis B & D infection | ACLF | no | no |
| 8 | 65 | M | 4 | 30 | no | no | ASH | CLF | no | no |
| 11 | 67 | F | 3 | 20 | yes | no | Polycystic kidney and liver disease | CLF | yes | no |
| 12 | 56 | M | 3 | 19 | no | no | NASH | CLF | no | no |
| 13 | 35 | M | 3 | 20 | yes | no | Polycystic kidney and liver disease | CLF | no | no |
| 14 | 35 | M | 3 | 37 | no | no | SLE, autoimmune hepatitis | CLF | yes | yes |
| 15 | 44 | M | 2 | 28 | no | yes | Hepatitis B & D infection | CLF | no | no |
| 16 | 40 | F | 3 | 30 | no | yes | Cryptogenic cirrhosis | ALF | no | no |
| 17 | 64 | M | 3 | 23 | no | no | NASH/ ASH, HCC | CLF | no | no |
| 19 | 56 | M | 2 | 10 | no | no | HCC, hepatitis B infection | CLF | no | no |
| 20 | 39 | F | 3 | 39 | no | Yes | NASH | ACLF | no | no |
| 21 | 67 | M | 3 | 29 | no | yes | PSC | ACLF | yes | no |
| 22 | 63 | F | 3 | 37 | yes | no | PBC/ NASH, autoimmune hepatitis | CLF | yes | no |
| 24 | 39 | M | 3 | 28 | yes | no | ITBL (Re-LTX) | ACLF | no | no |

ACLF: acute on chronic liver failure; ALF: acute liver failure; ASA: American Society of Anesthesiologists physiology score; ASH: alcoholic steatohepatitis; CLF: chronic liver failure; HCC: hepatocellular carcinoma; HE: hepatic encephalopathy; IgG4-related SC: Immunoglobin G4 related sclerosing cholangitis; ITBL: ischemic-type biliary lesions; LTX: liver transplantation; MELD: model for the end stage of liver disease; NASH: Non-alcoholic steatohepatitis; PBC: primary biliary cholangitis; PSC: Primary sclerosing cholangitis; SLE: Systemic lupus erythematosus; yrs.: years
