# Supplementary material for: Evaluation of dynamic cerebrovascular autoregulation during liver transplantation
Source: PLoS One. 2024 Jul 26;19(7):e0305658. doi: 10.1371/journal.pone.0305658 (PMC11280153; doi:10.1371/journal.pone.0305658)
Supplement: S1 File — (PDF) [file pone.0305658.s002.pdf]

PatID	Alter	Geschlecht 0=m, 1=w	Größe	Gewicht	APCHE II	SOFA	MELD
1	31	0	176	83	--	--	6
2	57	1	154	51	--	8	30
3	56	1	176	56	--	--	19
4	59	1	166	69	--	14	14
5	64	0	176	140	--	--	10
6	66	0	185	60	--	13	11
7	38	0	160	63	--	9	34
8	65	0	180	79			30
9	OP Abbruch						
10	Keine Messung möglich						
11	67	1	163	52		6	20
12	56	0	188	92			19
13	35	0	195	75		7	20
14	35	0	186	60			37
15	44	0	185	113		13	28
16	40	1	178	70			30
17	64	0	162	61		6	23
18	tech. Messfehler						
19	56	0	174	106		7	10
20	39	1	163	80		10	39
21	67	0	170	75		10	29
22	63	1	160	100		11	37
23	Keine Messung möglich						
24	39	0	160	60		9	28

Dialyse (0=nein, 1=ja)	Bilirubin	Quick	GOT	GPT	GLDH	Präparation			
						MAP	CI	HF	CBFVmean
0	0,6	96	20	34	168	77	3,8	55	--
0	14,3	24	89	23	699	64	5,1	81	42
0	8,3	46	144	56	267	72	3,4	58	--
0	2,4	55	55	29	388	75	4,8	73	57
0	1,1	78	46	17	254	64	7	95	71
0	2	78	49	27	138	70	4,6	82	76
0	41,6	38	170	60	155	79	4,7	72	50
0	8	31	77	116	367	85	8,2	79	39
1	1,2	62	413	833	800	63	4,1	64	39
0	4,2	46	52	49	315	98		75	42
1	1,1	96	35	42	300	75	9,1	64	68
0	29	13	180	168	336	77	4,7	67	33
0	11	23	55	40	224	86	5,2	86	36
0	23	23	386	1082	297	71	4,1	85	50
0	9,8	58	127	67	366	75	3,4	112	49
0	2,2	81	41	19	264	76	3,5	57	56
0	23,7	38	112	6	167	82	7,4	98	55
0	6,6	39	31	6	142	99	3,3	78	38
1	19,1	43	84	16	229	64	6,8	94	20
1	7,8	69	100	86	36	101	3,1	69	28

paCO2	Sevo (MAC)	Arterenol ml/h	coh_vlf_p	coh_lf_p	coh_hf	gain_vlf_p	gain_lf_p	gain_hf_p
39	1,7	0,5	0,02	0,02	0,06	0,28	0,25	0,32
43	0,7	0,1	0,02	0,01	0,02	0,15	0,09	0,11
39	1	5,5	0	0,02	0,03	0,07	0,11	0,01
36	0,8	18	0	0,01	0,03	0,06	0,08	0,18
40	1	6	0,04	0,12	0,49	1,19	0,83	1,28
37	0,8	14	0	0,01	0,02	0,06	0,12	0,2
33	0,7	1	0,07	0,06	0,02	0,11	0,1	0,03
59	1	15	0,05	0,07	0,28	0,53	0,82	1,45
41	0,8	2,1	0,01	0,03	0,04	0,28	0,58	0,57
37	0,5	0	0	0	0	0,02	0,01	0,02
45	0,7	12,2	0	0	0	0,04	0,04	0,06
30	0,8	9,2	0	0	0	0,02	0,02	0,05
44	0,7	8,2	0,01	0	0,01	0,05	0,04	0,06
39	0,6	12,1	0	0,04	0,02	0,24	0,24	0,14
38	0,7	7,1	0,02	0,08	0,3	0,05	0,19	0,7
			0,02	0,01	0,02	0,12	0,05	0,08
44	0,9	5,1	0	0	0,01	0,19	0,12	0,16
40	1	15,1	0	0	0	0,03	0,04	0,05
41	1	3	0	0	0	0,01	0,02	0,02
41	0,6	9	0,03	0,01	0,02	0,26	0,12	0,22
41,3	0,9	10	0,03	0,01	0,02	0,01	0,01	0,04

				anhepatisch					
phase_vlf_	phase_lf_	phase_hf_	Dauer	MAP	CI	HF	CBFVmean	paCO2	Sevo (MAC)
	-6,4	-12	124	77	2,6	75	--	35	2,4
18,78	-9,18	-6,68	105	56	3,3	70	36	37	0,9
12	14,91	-8,79	55	73	3,8	63	--	32	1
0,84	-19,8	1,65	38	74	3,3	99	85	35	1
7,65	-8,77	2,15	24	63	7,9	96	90	33	0,9
	-22,35	-33,2	120	72	3,3	143	41	38	0,9
85,28	-16,27	-25,67	55	61	4,1	81	69	31	0,7
122,76	52,43	-82,25	15	65	5,8	94	33	40	1
81,87	2,73	11,38	28	48	3,4	69	21	31	1
132,55	21,49	-9,4	58	85		91	46	40	0,7
64,06	6,92	-3,64	42	58	9,7	103	48	37	0,9
158,5	8,77	86,37	24	69	3,5	85	35	27	0,8
57,5	36,64	33,77	50	58	4,9	97	39	40	1,1
	2,07	-20,38	35	71	2,9	78	54	39	0,7
	-5,82	-4,63	12	57	3,3	135	41	35	0,8
	12,91	11,97							
	-16,7	-17,44	59	80	4,4	70	84	49	1
18,42	1,07	-12,14	11	90	4,3	89	69	34	0,8
29,73	-6,24	-2,07	47	82	2,9	77	20	37	0,8
16,18	50,96	-6,82	37	90	6,2	101	26	36	0,7
104,15	73,05	54,66	101	86	3,3	110	43	38,8	0,8

Arterenol	coh_vlf_	coh_lf_	coh_hf_	gain_vlf_	gain_lf_	gain_hf_	phase_vlf_	phase_lf_	phase_hf_	Dauer
10	0,02	0,01	0,04	1,67	1,09	0,86	80,51	78,84	-14,88	68
3,1	0,01	0,04	0,05	0,05	0,04	0,02	81,49	62,97	3,54	27
5	0	0	0,01	0,25	0,27	0,3	11,48	39,47	9,37	51
30	0,14	0,22	0,09	0,13	0,17	0,19		-15,19	5,23	43
10	0,02	0,03	0,06	0,75	1,32	0,94	60,94	-0,4	-17,07	30
58	0,01	0	0,03	1,52	0,77	0,43	95,03	-32,06	-2,84	28
3	0	0,11	0,01	0,24	0,63	0,22	135,55	-33,1	6,04	29
22	0,04	0,09	0,16	1,95	2,42	2,79	49,79	10,22	-3,15	25
3	0	0,02	0,04	1,34	2,7	1,46	86,88	59,02	11,88	22
1	0,05	0,03	0,02	3,15	1,51	0,74		21,03	-3,4	20
9,1										37
6,1	0	0	0	0,02	0,01	0,02	4,11	-7,01	20,36	30
5,1	0	0,02	0,01	2,79	3,04	2,06	20,61	20,14	24,91	43
13,1	0,01	0,07	0,07	3,5	2,91	2,38	23,36	-14,8	-19,52	32
16	0	0,11	0,46	0,06	0,39	1,08	20,51	-15,4	-6,92	26
6,1	0,03	0,19	0,14	1,44	1,59	1,47	34,72	2,34	-13,47	37
12,1	0	0,02	0	0,06	0,15	0,07		-15,33	48,98	47
5	0,05	0,03	0,02	0,29	0,15	0,13	121,78	12,35	-31,62	61
12	0	0	0,02	0,03	0,08	0,11	31,76	-10,66	0,71	70
10	0	0,01	0,01	0,03	0,05	0,05		-2,9	17,88	116

Reperfusion

MAP	CI	HF	CBFVmean	paCO2	Sevo (MAC)	Arterenol	coh_vlf_	coh_lf_r	coh_hf_
76	2,9	100	--	44	2,1	10	0,3	0,58	0,6
74	5,7	79	62	43	0,8	12,1	0,11	0,01	0,1
72	2	60	--	41	0,8	14	0,05	0,02	0,05
	5,2	92	83	50	0,7	18	0,08	0,07	0,07
58	5,4	89	91	34	1	18	0,01	0,02	0,13
68	2,5	104	87	44	0,8	62	0,03	0,11	0,23
40	5	67	62	37	0,7	3	0	0,03	0
70	2,5	96	41	42	1	15	0	0	0,01
30	3,8	56	22	41	1	3	0,01	0,02	0,06
70		89	58	43	0,6	0,1	0	0	0
23	3,4	88	46	40	1	6,1	0	0	0,01
62	3,3	86	33	30	0,7	7,1	0,02	0,02	0,02
78	4,2	110	52	45	1	6,1	0	0	0
78	2,1	88	87	53	0,8	9	0,01	0	0,09
54	3,7	109	54	36	0,8	14	0,01	0,14	0,37
69	3,2	81	76	53	1	16	0,01	0,03	0,07
64	4,6	82	58	36	0,7	18	0	0	0
50	2,8	74	28	43	0,7	5	0	0	0,02
53	2,6	90	40	45	0,6	15	0,01	0	0,03
41	2,4	63	43	37,6	0,9	10	0,02	0,02	0,18

gain_vlf_	gain_lf_r	gain_hf_	phase_vl	phase_lf_	phase_h	Dauer	Outcome lenght of ICU stay d
0,6	0,77	0,74		-15,62	-10,84	133	2
0,27	0,02	0,33	6,88	-18,08	-23,59	110	19
0,08	0,03	0,15	95,19	-22,8	7,34	27	3
0,13	0,08	0,21	45,87	-2,37	7,96	40	62
1,46	0,89	1,12		-22,15	7,59	60	2
0,88	1,23	0,93		9,76	-5,85	16	54
0,32	0,7	0,23	16,07	-48,6	37,23	154	2
0,08	0,09	0,4	79,75	-4,34	-27,8	84	2
1,06	1,57	1,02	20,4	30,97	-8,14	30	5
0,05	0,04	0,18		87,29	-19,38	80	1
0,08	0,47	0,71	46,17	19,08	45,39	35	2
1,69	5,32	2,41	66,27	22,77	21,8	31	18
0,05	0,2	0,4	15,89	-8,49	19,79	31	5
1,07	1	0,76	21,89	-18,77	-0,98	36	1
0,27	0,99	1,71	24,3	-4,6	-10,98	56	4
1,94	7,85	7,58	83,54	-0,75	-5,32	44	2
0,01	0,04	0,07	73,12	14,38	-3,79	16	4
0,13	0,14	0,27	157,33	45,85	16,58	57	10
3,19	1,79	1,19		55,22	9,69	70	6
2,54	1,19	1,27		-11,64	-19,63	30	2

length of hospital stay d	CAM-ICU innerhalb der ersten 4 d (ICU) 0=negativ, 1=positiv, 2=nicht erhebbar	Blutprodukte intraoperativ EK
13	0	0
35	0	3
36	0	0
94	0	8
18	0	8
54	2	28
15	0	4
13	0	5
26	1	2
13	0	5
16	0	4
41	1	2
88	0	0
38	0	4
14	0	2
8	0	0
22	0	10
39	1	12
31	1	9
11	0	8

		Follow up 1 Jahr	Mortalität innerhalb eines Jahres	Re-LTX	Autoimmun
FFP	TK	0=nein, 1=ja	0=nein, 1=ja	0=nein, 1=ja	0= nein, 1= ja
3	0	1	0	0	1
2	0	1	0	0	0
0	0	1	0	0	1
14	2	0	1	0	0
6	0	1	0	0	0
34	2	0	1	0	1
6	0	1	0	1	0
10	2	1	0	0	0
6	0	2	0	0	0
6	0	1	0	0	0
10	2	1	0	0	0
4	1	0	1	0	1
0	0	1	0	0	0
11	0	1	0	0	0
7	0	1	0	0	0
0	0	1	0	0	0
8	0	1	0	0	0
20	2	1	0	0	1
8	0	1	0	0	1
					0
11	2	1	0	0	0

Ursache	Onset<br>0= chronisch, 1= akut, 2= ACLF
HCC bei Leberadenomatose, MODY 3	0
kryptogene Leberzirrhose	2
PBC/ Autoimmunhepatitis	0
NASH	0
NASH	0
IgG4 ass. Cholangitis	0
Hep B mit Delta Virus	2
ASH	0
Polyzystische Nieren- u. Lebererkrankung	0
NASH	0
Polyzystische Nieren- u. Lebererkrankung	0
Autoimmunhepatitis, Syst. Lupus	0
HBV/ HDV	0
kryptogen	1
ASH/ HCC	0
HCC, Z.n. HBV	0
NASH	2
PSC	2
PBC/ NASH	0
HCC, Z.n. HBV	0
Re-LTX bei Cholangiosepsis; bei Hep B mit Delta Virus	0

Hepatische Enzephalopathie<br>0= nein, 1= ja	OP-Technik<br>0= Cavaersatz, 1=Piggyback
0	0
0	0
0	1
1	0
1	1
0	1
0	0
0	1
0	0
0	1
0	0
0	1
1	1
0	1
0	1
0	1
0	1
0	1
0	1
0	0
0	0

Flush
0= Blut, 1= Sterofundin
0
0
1
0
1
1
0
0
0
0
0
1
1
1
1
1
1
1
1
0
1
