# Supplementary material for: Evaluation of dynamic cerebrovascular autoregulation during liver transplantation
Source: PLoS One. 2024 Jul 26;19(7):e0305658. doi: 10.1371/journal.pone.0305658 (PMC11280153; doi:10.1371/journal.pone.0305658)
Supplement: Attachment — Submitted filename: PLOS ONE_response to reviewer_2024_01_24.pdf [file pone.0305658.s003.pdf]

Point-by-point response on submission PONE-D-23_26201

Dear Academic Editor Prof. Boteon,
dear Reviewers,

we are most grateful for your detailed review of our manuscript and for your expert questions and suggestions with regard to our data. Having thoroughly studied your assessments, we have revised the manuscript substantially. We are convinced that the quality of the manuscript has significantly improved. Please find below a point-by-point response to your concerns. All changes to the original manuscript are tracked and have been highlighted in yellow in case of a supplement or by crossed out words in case of a deletion.

Thank you very much for the efforts to improve the manuscript.

Yours sincerely
Eva-Verena Griemert

Editor comments

1. Please ensure that your manuscript meets PLOS ONE's style requirements, including those for file naming.

   We checked the PLOS ONE's style requirements and revised the manuscript accordingly.

2. Please ensure that you refer to Figure 2 in your text as, if accepted, production will need this reference to link the reader to the figure.

   Thank you for indicating the missing reference to Figure 2 in the text, which we have integrated.

3. Please include captions for your Supporting Information files at the end of your manuscript, and update any in-text citations to match accordingly.

   We included captions for the supporting information file at the and of the manuscript. The in-text citations were checked for matching accordingly.

Review comments to the Author

Reviewer #1: Dear Editor and authors,

I would like to thank you for the opportunity to review this manuscript. Investigation of cerebral autoregulation during Liver transplantation is an interesting theme. The manuscript in itself is relatively well written with an easy to follow introduction, methods, results, and discussion section. However, I have some issues and comments which must be addressed. I have spent a lot of time investigating the reliability and validity of Mx and the corresponding indices. Based on a systematic review (https://pubmed.ncbi.nlm.nih.gov/34617816/), we concluded that the methodology on how to calculate Mx varies throughout the literature, which limits comparisons between studies. This is exemplified in this observational study (https://pubmed.ncbi.nlm.nih.gov/34173717/) where different block and epoch lengths are investigated. In this study other factors seems to influence the reliability of Mx. Especially the idea of dichotomizing Mx seems flawed, since multiple studies showed that Healthy volunteers on average have an Mx of above 0.3 or even 0.45 which is recommended by Schmidt et al. [1–3]. Furthermore, the validity is questionable (https://pubmed.ncbi.nlm.nih.gov/35343649/; https://pubmed.ncbi.nlm.nih.gov/36008917/). Thus, I do not believe Mx should be used to assess cerebral autoregulation. Preliminary results (unfortunately unpublished) provide somewhat better reliability for Transfer Function Analysis. We have developed an R-package which can calculate TFA (https://cran.r-project.org/web/packages/clintools/index.html). To get started with TFA I would suggest looking into https://pubmed.ncbi.nlm.nih.gov/26782760/ and https://pubmed.ncbi.nlm.nih.gov/35962478/. Furthermore Jürgen Claassen have provided a great overview for the current knowledge of cerebral autoregulation which is rather long, but for some very interesting (https://pubmed.ncbi.nlm.nih.gov/33769101/). TFA has its own issues, but I do not have any data concluding that TFA should not be used as a measure of cerebral autoregulation.

In conclusion, I do not believe Mx can be dichotomized and it should not be used as a measure for cerebral autoregulation.

References:

1. Ortega-Gutierrez S, Petersen N, Masurkar A, Reccius A, Huang A, Li M, et al. Reliability, asymmetry, and age influence on dynamic cerebral autoregulation measured by spontaneous fluctuations of blood pressure and cerebral blood flow velocities in healthy individuals. J Neuroimaging. 2013/04/24. 2014;24:379–86.

2. Reinhard M, Wehrle-Wieland E, Roth M, Niesen WD, Timmer J, Weiller C, et al. Preserved

dynamic cerebral autoregulation in the middle cerebral artery among persons with migraine. Exp Brain Res. 2007/02/07. 2007;180:517–23.
3. Yam AT, Lang EW, Lagopoulos J, Yip K, Griffith J, Mudaliar Y, et al. Cerebral autoregulation and ageing. J Clin Neurosci. 2005;12:643–6.

Response to 1.1
Dear Prof. Olsen,
thank you very much for spending so much time in the improvement of our manuscript, especially regarding your feedback to Mx. We addressed this topic and recalculated all data with the R-package and double-checked it with the help of our local statistician Robert Kuchen.
Our working group started clinical autoregulation measurements with the static procedures following the first dynamic method leading to the ICM+ and the index Mx. While proceeding measurements in the operation theatre a MATLAB program was written including the time (Mx) and the frequency domain (TFA). Our manuscript was written with Mx because our working group felt more familiar with this parameter, despite the already known issues. Therefore, we recalculated the data using R-package. Fortunately, the results in R and theses of our MATLAB were similar which led to confirmation of our previous calculations. Accordingly, all sections except for introduction were mainly re-written, including the time and frequency domain analyses. In short, all dCA calculations were repeated an cross-checked by our statistician.
We totally agree, that dCA is rather a continuous than a binary measure and highlighted this aspect in particular in the discussion.
We thank you and your group very much for providing the R program.

Reviewer #2: Authors present a cerebral autoregulation analysis in patients undergoing liver transplant. Data are acquired in the different phases of surgery.
The paper is interesting and original. However scanty data and analyses are presented and the work should be improved in this sense.
2.1 Introduction: there is lack of reference to some previous works analyzing correlation of MAP and MCBv in surgery.

Response to 2.1
Dear Reviewer#2,
we thank you very much for your comments, which have definitively improved the manuscript. We amended the introduction by adding references as recommended.

2.2 The use of correlation instead of other index of cerebral autoregulation (E.g. Autoregulation Index: Tiecks et al., Stroke. 1995 Jun;26(6):1014-9. doi: 10.1161/01.str.26.6.1014.; Gelpi et al., Auton Neurosci. 2022 Jan;237:102920. doi: 10.1016/j.autneu.2021.102920. or Transfer function analysis Panerai et al.,J Cereb Blood Flow Metab. 2023 Jan;43(1):3-25. doi: 10.1177/0271678X221119760..) should be better motivated.

Response to 2.2

We worked on this topic intensively. In sum, we fully recalculated the dCA calculations with a standardized R-package (see reviewer #1) according to the recommendation of Panerai. Considering the published results of the dCA measurement in the time domain, we decided to show all parameters of dCA regarding the time and frequency domain analyses.

2.3 Methods:

How did you synchronize the CBFV and AP signals? If signals were not properly synchronized the correlation could differ.

Response to 2.3

The signal of the ABP were analogue transferred directly after amplifying to the Doppler Box were both signals were digitalized with a sampling rate of 100 MHz. To clarify the following sentence was added in the “dCA measurement” paragraph: *“Invasive arterial blood pressure was measured in the radial artery (M10006B/MP70, Philips Medical Systems, Eindhoven, Netherlands). The analogue curves of ABP and CBv were synchronically recorded and digitalized with a sampling rate of 100 Hz by the Doppler Box©. Before analysis of the dCA, the recorded curves were visually viewed and artifacts were removed from the recordings.”*

2.4 For consistency with the other works evaluating CA I would suggest you to use "CBv" instead of "CBFV" and "MCBv" in place of "MCBFV". (See the White paper on CA update, Panerai et al., J Cereb Blood Flow Metab. 2023 Jan;43(1):3-25. doi: 10.1177/0271678X221119760.)

Response to 2.4

As recommended, we replaced the acronyms “CBFV” by “CBv” and “MCBFV” by “MCBv” and “CA by “dCA” according to the CARNet white paper.

2.5 Why did you use 6-seconds windows average on the signals and not provide first a beat-to-beat estimation of the mean CBv and mean AP (MAP)? A reference would be

needed.

Response to 2.5

We used the calculation in the time domain according to the working group of M. Czosnika and P. Smielewski who introduced this calculation in many studies and already in the device ICM+. In the revised version the time window was changed to 3 seconds according to the R-package of the CARNet. We included the reference respectively.

2.6 How long were the recordings? How many windows were calculated and how did authors obtained a single value for acquisition phase?

Please clarify and consider to use a figure to explain the computations.

Response to 2.6

The measurement was obtained over the whole phase of LTX and the average of the Mxa values and the TFA were adopt over the whole phase. The windows, in the actual version 1 minute instead of 3, is a moving window, so the correlation will be done all second for the minute behind, therefore the windows are not countable. This method, also the long measurement timeframe, are well described and used in OR and on the ICU.

2.7 Line 139: define the MELD score and how it is calculated.

Response to 2.7

We have defined the MELD score and explained the parameters on which the calculation is based.

2.8 A table resuming demographic and clinical characteristics in terms of average value in the acquired population would improve the work.

Response to 2.8

We have expanded Table 1 with demographic and clinical characteristics. Additionally, a detailed table in the supplement (S1 Table) provides corresponding information for each individual patient.

2.9 Paper evaluating CA normally evaluate it from series of mean arterial pressure (MAP) and mean cerebral blood velocity (MCBv) extracted on a beat-to-beat basis or in opportune windows.

That should be done for the three different experimental phases and the classical markers of mean and variance (or similar) of MAP and MCBv should be given.

Response to 2.9

The beat-to-beat extraction for the parameter MAP and MCBv were done, but not presented in the paper. Now, we presented these parameters at the beginning of the “Result – Primary

Outcome" and compare these parameters to the fully reanalyzed dependent parameters with ANOVA.

2.10 Furthermore, the statistical analysis is not completely clear. Which conditions were compared with a t-test?
Why not performing an ANOVA among the three phases of intervention to observe if in the overall population there were differences in the experimental phases?
The significance of the Pearson's correlation coefficient should be given. Anyway, the significance of the coupling should be tested via a surrogate analysis to check if the real value of correlation is different from a random coupling (see the one proposed in Bari et al. BPSC 2021, https://doi.org/10.1016/j.bspc.2021.102735, in similar conditions, or use e.g. an Iteratively-refined amplitude adjusted Fourier transform-based method(Schreiber 1996, Phys. Rev. Lett. 77 635–8 DOI:10.1103/PhysRevLett.77.635)

Response to 2.10

Thank you for this relevant comment. After recalculation of the data the phases were compared using ANOVA in regard of the time domain (Mxa) and the frequency domain (TFA) in the very low, low and high frequency range. All data were double-checked by our local statistician.

2.11 Please pay attention that Raw data, e.g. time series or recordings are not declared as fully available as requested by the journal.

Response to 2.11

Due to very strict data protection regulations by our University Medical Center, we would prefer to only provide the raw data on request. Based on the fact of a very specific and small patient population as well as a single center investigation, we have to ensure the compliance to our local regulations. We hope, this procedure obtains your approval.
