# Supplementary material for: Evaluation of dynamic cerebrovascular autoregulation during liver transplantation
Source: PLoS One. 2024 Jul 26;19(7):e0305658. doi: 10.1371/journal.pone.0305658 (PMC11280153; doi:10.1371/journal.pone.0305658)
Supplement: Attachment — Submitted filename: Response to Reviewers.docx [file pone.0305658.s004.docx]

**Response to Reviewers**

**Point-by-point response on submission PONE-D-23-26201R1**

Dear Editor Prof. Boteon,

dear Referees,

thank you very much for your second review of the revised manuscript according to your first given expert questions and suggestions. We thoroughly studied your second assessments and have revised the manuscript accordingly. Below, we will explain how we have implemented your suggestions and comments.

We are convinced that the quality of the manuscript has again significantly improved and hope it will come up to your expectations comprehensively. Please find below a point-by-point response to your concerns. All changes to the original manuscript are tracked and have been highlighted in YELLOW.

Thank you very much for the efforts to improve the manuscript.

Yours sincerely

Eva-Verena Griemert

**Reviewer's Responses to Questions**

**Comments to the Author**

**1. If the authors have adequately addressed your comments raised in a previous round of review and you feel that this manuscript is now acceptable for publication, you may indicate that here to bypass the “Comments to the Author” section, enter your conflict of interest statement in the “Confidential to Editor” section, and submit your "Accept" recommendation.**

**Reviewer #1:** (No Response)

**Reviewer #2:** All comments have been addressed.

**2. Is the manuscript technically sound, and do the data support the conclusions?**
The manuscript must describe a technically sound piece of scientific research with data that supports the conclusions. Experiments must have been conducted rigorously, with appropriate controls, replication, and sample sizes. The conclusions must be drawn appropriately based on the data presented.

**Reviewer #1:** Partly

**Reviewer #2:** Yes

**3. Has the statistical analysis been performed appropriately and rigorously?**

**Reviewer #1:** N/A

**Reviewer #2:** Yes

**4. Have the authors made all data underlying the findings in their manuscript fully available?**
The [PLOS Data policy](http://www.plosone.org/static/policies.action#sharing) requires authors to make all data underlying the findings described in their manuscript fully available without restriction, with rare exception (please refer to the Data Availability Statement in the manuscript PDF file). The data should be provided as part of the manuscript or its supporting information, or deposited to a public repository. For example, in addition to summary statistics, the data points behind means, medians and variance measures should be available. If there are restrictions on publicly sharing data—e.g. participant privacy or use of data from a third party—those must be specified.

**Reviewer #1:** No

**Reviewer #2:** No

**5. Is the manuscript presented in an intelligible fashion and written in standard English?**
PLOS ONE does not copyedit accepted manuscripts, so the language in submitted articles must be clear, correct, and unambiguous. Any typographical or grammatical errors should be corrected at revision, so please note any specific errors here.

**Reviewer #1:** Yes

**Reviewer #2:** Yes

**6. Review Comments to the Author**

**Reviewer #1:**

Dear authors
I would like to thank the reviewer for improving the manuscript. Unfortunately, we have recently published an article discussing why Mx and the derived indicies (Mxc, Mxa and Mxa) should not be used (<https://physoc.onlinelibrary.wiley.com/doi/10.1113/EP091327>). I suggest removing Mx from the manuscript and focus on TFA.

**Response to Reviewer #1:**

Dear Prof. Olsen,

thank you very much for spending so much time in the improvement of our manuscript, especially regarding your feedback to Mxa. Following your extensive publications, we have focused on the TFA and removed Mxa from the manuscript. As a result, we adapted the text and modified table 3 accordingly. Additionally, the original “Figure 1” has been removed and the numbering of the following figures has been adjusted accordingly. The individual courses mentioned in the discussion, which were based on observations of Mxa, have been completely removed for this reason.

**Reviewer #2:** The paper has been improved and the questions raised by the reviewers have been addressed. I would suggest authors to further improve the clarity of figures better describing in figure captions the meaning of the symbols used for statistical significance (e.g. * means p<0.05 reperfusion vs preparation phase), or using a line between boxplots resulting significantly different instead of a single symbol.

**Response to Reviewer #2:**

Dear Reviewer #2,

Thank you very much for your feedback on our manuscript. We have implemented your comments. By improving the captions and legends, uncertainties may now have been clarified. Furthermore, we revised the figure style. Outliners are now indicated as small dots to prevent any misunderstandings regarding significance levels.

**Additional comments:**

During the revision and implementation of your helpful comments, we recognized the need for a more detailed presentation of the statistical analysis. Consequently, we have enhanced the Statistical Analysis section with detailed information. Additionally, Table 3 has been expanded to include both mean + SD and median (range), as well as NA values for phase and gain across the three frequency bands. Upon reviewing the results, we noticed that the estimation algorithm of the mixed-effect ANOVA did not converge in two cases. We replaced these two mixed-effect models with generalized estimating equations, which slightly altered the p-values. However, these changes did not generally affect the results. Furthermore, we considered it relevant to conduct post-hoc pairwise comparisons to test for differences between two specific phases. To address the issue of multiple comparisons, Sidak corrections were applied. The resulting p-values are listed in Table 4. Consequently, there have been slight changes to the p-values in the conclusions provided in the primary outcome section.

**7. PLOS authors have the option to publish the peer review history of their article (**[**what does this mean?**](https://journals.plos.org/plosone/s/editorial-and-peer-review-process#loc-peer-review-history)**). If published, this will include your full peer review and any attached files.**
If you choose “no”, your identity will remain anonymous but your review may still be made public.
Do you want your identity to be public for this peer review? For information about this choice, including consent withdrawal, please see our [Privacy Policy](https://www.plos.org/privacy-policy).

**Reviewer #1:** Yes: Markus Harboe Olsen

**Reviewer #2:** No
